# Comprehensive analysis of the prognostic signature and tumor microenvironment infiltration characteristics of cuproptosis-related lncRNAs for patients with colon adenocarcinoma

**DOI:** 10.3389/fonc.2022.1007918

**Published:** 2022-09-23

**Authors:** Guoliang Cui, Jinhui Liu, Can Wang, Renjun Gu, Manli Wang, Zhiguang Sun, Fei Wei

**Affiliations:** ^1^ Department of Gastroenterology, The Second Affiliated Hospital of Nanjing University of Chinese Medicine, Nanjing, China; ^2^ Department of Gynecology, The First Affiliated Hospital of Nanjing Medical University, Nanjing, China; ^3^ Department of Colorectal Surgery, Jiangsu Province Hospital of Chinese Medicine, Affiliated Hospital of Nanjing University of Chinese Medicine, Nanjing, China; ^4^ School of Traditional Chinese Medicine and School of Integrated Chinese and Western Medicine, Nanjing University of Chinese Medicine, Nanjing, China; ^5^ The First Clinical Medical College, Nanjing University of Chinese Medicine, Nanjing, China; ^6^ Department of Physiology, School of Medicine & Holistic Integrative Medicine, Nanjing University of Chinese Medicine, Nanjing, China

**Keywords:** colon adenocarcinoma, cuproptosis, prognosis, tumor immune microenvironment, bioinformatics

## Abstract

**Background:**

Cuproptosis, a newly described method of regulatory cell death (RCD), may be a viable new therapy option for cancers. Long noncoding RNAs (lncRNAs) have been confirmed to be correlated with epigenetic controllers and regulate histone protein modification or DNA methylation during gene transcription. The roles of cuproptosis-related lncRNAs (CRLs) in Colon adenocarcinoma (COAD), however, remain unknown.

**Methods:**

COAD transcriptome data was obtained from the TCGA database. Thirteen genes associated to cuproptosis were identified in published papers. Following that, correlation analysis was used to identify CRLs. The cuproptosis associated prognostic signature was built and evaluated using Lasso regression and COX regression analysis. A prognostic signature comprising six CRLs was established and the expression patterns of these CRLs were analyzed by qRT-PCR. To assess the clinical utility of prognostic signature, we performed tumor microenvironment (TME) analysis, mutation analysis, nomogram generation, and medication sensitivity analysis.

**Results:**

We identified 49 prognosis-related CRLs in COAD and constructed a prognostic signature consisting of six CRLs. Each patient can be calculated for a risk score and the calculation formula is: Risk score =TNFRSF10A-AS1 * (-0.2449) + AC006449.3 * 1.407 + AC093382.1 *1.812 + AC099850.3 * (-0.0899) + ZEB1-AS1 * 0.4332 + NIFK-AS1 * 0.3956. Six CRLs expressions were investigated by qRT-PCR in three colorectal cancer cell lines. In three cohorts, COAD patients were identified with different risk groups, with the high-risk group having a worse prognosis than the low-risk group. Furthermore, there were differences in immune cell infiltration and tumor mutation burden (TMB) between the two risk groups. We also identified certain drugs that were more sensitive to the high-risk group: Paclitaxel, Vinblastine, Sunitinib and Elescloml.

**Conclusions:**

Our findings may be used to further investigate RCD, comprehension of the prognosis and tumor microenvironment infiltration characteristics in COAD.

## Introduction

Colorectal cancer (CRC) is the third most frequent cancer and the second major cause of cancer-related death worldwide (1, 2). In 2018, 1.8 million new instances of CRC were diagnosed, with over 800,000 deaths (3). The pathogenesis involves a chronic process, including precancerous lesions, activation of tumor stem cells, accumulation of genetic and epigenetic changes (4). CRC is a heterogeneous disease with widespread chromosomal instability and microsatellite instability (5). CRC morbidity and mortality are declining in most developed countries due to early screening and prevention of early risk factors (6). However, the situation of CRC in developing countries is still very serious (7). The pathogenesis of CRC involves a series of multi-step changes, including histological, morphological, and genetic changes (8, 9). Unhealthy diet, obesity, smoking and alcohol consumption are considered risk factors for CRC (10, 11). The 5-year overall survival (OS) rate of localized and regionalized CRC patients is impressively high, but decreases to 14% once metastasis occurs (12). In the past decade, immunotherapy has become a hot topic in refractory solid tumors due to its long-term response. Immunotherapy significantly inhibit the progression of advanced malignant tumors and prolong the survival of patients, which brings hope to CRC patients (13). Colon adenocarcinoma (COAD) is the ordinary histological subtype of CRC, therefore, it is of great value to explore a new prognosis assessment protocol and to establish a predictive signature for immunotherapy and immune microenvironment of COAD.

In recent years, regulatory cell death (RCD) plays an important role in maintaining normal homeostasis of body development and inhibiting rapid proliferation of tumor cells, which is considered as a new direction of tumor therapy (14, 15). In recent years, the most widely studied types of RCD are apoptosis, pyroptosis, necroptosis and ferroptosis (16, 17). Different from the known mechanism of cell death, Tsvetkov et al. found that cuproptosis is a new form of cell death, namely the existence of a copper-dependent, regulated cell death in human cells (18). Cuproptosis relies on the effect of copper ions on mitochondrial tricarboxylic acid metabolism, resulting in abnormal aggregation of lipoacylated proteins and loss of iron-sulfur (Fe-S) cluster proteins, which leads to the proteotoxic stress response of tumor cells and cell death (18, 19). However, the mechanism of RCD and its role in tumor microenvironment have not been thoroughly studied, which may play a double-edged sword role in tumors (17, 20). On the one hand, inducing tumor cell death can cure tumors; On the other hand, when the inflammatory response caused by cell death reaches a certain level, many signaling pathways can be activated, leading to tumor progression (21). Therefore, it is of great significance to explore the role of RCD in tumors, and many studies have established RCD-associated prognostic models to assess prognosis and immune microenvironment (22–24). However, the clinical significance of cuproptosis and cuproptosis-associated prognostic model have not been reported, especially in COAD.

Long noncoding RNAs (lncRNAs) can regulate gene expression through epigenetic regulation, transcriptional regulation and post transcriptional regulation, so as to participate in a variety of biological processes such as tumor cell proliferation, differentiation and apoptosis (25). Therefore, lncRNAs are considered as promising biomarkers and potential therapeutic targets for the diagnosis and treatment of various diseases, including COAD (26). More and more attention has been paid to the role and molecular mechanism of regulating RCD-related lncRNAs in tumor pathology (27). Therefore, as a new type of RCD, the identification of lncRNAs related to cuproptosis is of great significance for understanding the pathogenesis of tumor and providing new targets for prevention and treatment.

Through bioinformatics research, we investigated the importance of cuproptosis-related lncRNAs (CRLs) and a prognostic signature based on CRLs was constructed in COAD. A risk score for each COAD patient might be determined based on cuproptosis-associated model, which can be used for prognosis assessment, immunological prediction, and mutation analysis. Our findings may be useful in determining the prognosis and therapy of patients with COAD.

## Methods and materials

### Download of data

The TCGA database was used to obtain the data of COAD RNA sequencing and clinical information. We collected data from 473 tumor samples and 41 healthy controls. From the previously published publications (18, 28), thirteen cuproptosis-related genes were obtained, including FDX1, LIPT1, LIAS, DLD, DBT, GCSH, DLST, DLAT, PDHA1, PDHB, SLC31A1, ATP7A, and ATP7B.

### Screening lncRNAs associated with cuproptosis

From the TCGA database, 1053 CRLs were identified using Pearson correlation analysis and a co-expression network was created based on the cutoff (Pearson R > 0.4 and *P* < 0.001) (29). Then, using univariate Cox regression analysis and forest maps, 49 CRLs with potential prognostic significance for COAD were identified. The “limma”, “pheatmap”, “reshape2”, and “ggpubr” programs were used to create heat maps and boxplots to show the differential expression of CRLs in COAD and normal tissues, with following criteria: |log_2_ fold change (FC)| >1 and false discovery rate (FDR) < 0.05.

### Consensus clustering analysis

To preliminarily understand the underlying the mechanism of the biological function of cuproptosis-related lncRNAs, The “ConsensusClusterPlus” package was used to construct a consensus cluster with 49 CRLs (K represents cluster count) (30). The cluster exhibited the best stability when K = 3 based on the similarity of expression levels of CRLs and the proportion of fuzzy clustering measures. As a result, 417 CRC patients were divided into three clusters: cluster 1 (n = 139), cluster 2 (n = 202), and cluster 3 (n = 76). The variations in survival, CRLs expression, and clinical characteristics were then compared among the three clusters. Immune checkpoint inhibitors co-expression (PD-L1, CTLA-4), immune cell content differences, and immunological score (including ESTMATE score, immune score and stromal score) were also investigated.

### Construction and evaluation of the prognostic model

All COAD patients were randomly assigned to one of two groups: training cohort or validation cohort (testing cohort and entire cohort). No significant difference was observed in three cohort for the clinical-pathological factors (**Supplementary Table 1**). In the training cohort, LASSO and multivariate Cox regression analysis were used to identify prognostic model based on CRLs. The risk score was calculated using the following formula: coef (lncRNA1) × expr (lncRNA1) + coef (lncRNA2) × EXPR (lncRNA2) +… + coef (lncRNAn) × expr (lncRNAn), coef stands for coefficient, coef (lncRNAn) stands for coefficient of survival linked lncRNA, and expr (lncRNAn) stands for lncRNA expression. Patients in the training set were separated into two groups based on their median risk score: high-risk and low-risk. For survival analysis, the R packages “survival” and “survminer” were used by Kaplan-Meier curve, and a ROC curve was plotted (31). Finally, we run the above analyses in a validation cohort to verify the predictive power of the results.

### Independent prognostic value assessment of the prognostic model

When paired with other clinical factors, univariate and multivariate Cox regression analysis were performed to determine whether risk score was an independent predictive factor in COAD patients (32).

### Cell culture

Three human colorectal cancer cell lines (Caco-2, HT-29, HCT116) were all purchased from the China Center for Type Culture Collection (CCTCC, Wuhan, China). The normal colon epithelial cell line (FHC) was obtained from the Cell Bank of Type Culture Collection of the Chinese Academy of Sciences (Shanghai, China). Caco-2, HT-29 cells, HCT116 and FHC were cultured in McCoy’s 5A, RPMI-1640, high-glucose DMEM medium (Gibco, Shanghai, China) respectively, which were supplemented with 10% fetal bovine serum (FBS, Gibco, Shanghai, China) and 1% antibiotics. All cells were incubated at 37°C with 5% CO_2_.

### Quantitative RT-PCR

Total RNA from the cell lines was isolated with TRIZOL reagent (Thermo Fisher Scientific, USA). Complementary DNA (cDNA) was synthesized and quantitative RT-PCR was performed using SYBR qPCR Master Mix (Vazyme, China). The relative expression of the target gene was analyzed using the 2^−ΔΔCT^ method and β-actin was chosen as the internal reference. The primer sequences are listed in **Supplementary Table 2**.

### Construction and evaluation of the nomogram

Based on risk scores and patient clinical information, a nomogram was created to predict 1-year, 3-year, and 5-year OS (33). The Hosmer-Lemeshow test was used to construct modified curves to show the agreement between the actual and anticipated outcomes. The accuracy of the nomogram was assessed using ROC curves (34).

### Gene set enrichment analysis

The Kyoto Encyclopedia of Genes and Genomes (KEGG) pathways were identified using gene set enrichment analysis (GSEA) (35). The GSEA website (http://software.broadinstitute.org/gsea/index.jsp) was utilized to identify gene-level enrichment. Based on the risk score model, COAD samples from the entire set were separated into high-risk and low-risk groups. The underlying biological functions of the two groups were compared. The molecular signature database (MSigDB, http://software.broadinstitute.org/gsea/msigdb/index.jsp) collection of annotated gene sets was chosen as a reference gene set in the GSEA software. The cut-off criterion was set at a notional *P* < 0.05. As a reference document, we use “c2.cp.kegg.v7.4.symbols.gmt.”

### Evaluation of the immune microenvironment

For immune score, stromal score, estimated score, each sample was evaluated using a “estimated” R-package. The proportion of immune to stromal components in the tumor microenvironment is represented by these scores. Pearson correlation coefficient approach was used to assess the correlations between immune score, stromal score, estimated score, and risk score. Based on TCGA RNA sequencing data, the CIBERSORT tool was utilized to quantify 22 types of immune cell components (36). TIMER, CIBERSORT, Cibersort-ABS, QUANTISEQ, MCPCOUNTER, XCELL, and EPIC databases were all used to calculate immune cell infiltration. The Pearson correlation coefficient approach was used to assess the link between immune cell infiltration and CRLs expression level, risk score. One-class logistic regression (OCLR) machine-learning algorithm was used to quantify the stemness of tumor samples by calculating cancer stem cell indices (37).

### Mutation analysis

TCGA provided mutation data (data category = copy number variation; “Maf” file). The top 20 mutant genes were visualized using waterfall diagrams created by the R software package “MAftools” (38). The tumor mutation burden (TMB), which is the number of somatic mutations per Megabyte genome sequence, can be used to identify patients who will respond better to immune checkpoint inhibitors (ICIs) (39). The differences of TMB between the two risk groups were investigated, as well as their correlation with risk score. The m^6^A-related genes and human leukocyte antigen (HLA) genes were compared between the two risk groups using the “limma” package (40).

### Drug sensitivity analysis

We compared the IC50 differences of the four chemotherapeutic drugs between the two risk groups using the R software package “PRROPHIC” (41). Using the R package “ggplot2,” researchers discovered a link between six CRLs and chemotherapeutic sensitivity (42). The relationship between CRLs expression and drug susceptibility was investigated using Pearson correlation analysis.

### Statistical analysis

The continuous variables in normal distribution are analyzed by Student’s t-test, which is presented as mean ± standard deviation, and the continuous variables in abnormal distribution are presented as median (range). A *p*-value less than 0.05 was considered as statistical significance.

## Results

### Screening of cuproptosis-related lncRNAs with prognostic value

**Figure 1:**
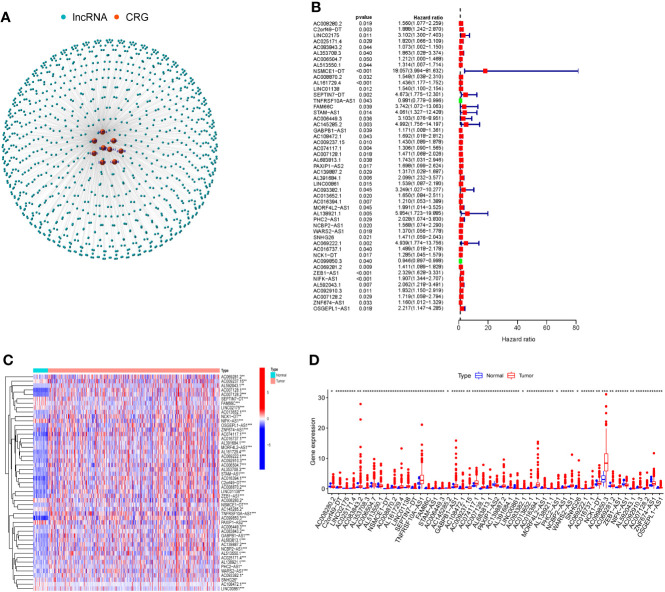
Cuproptosis-related lncRNAs (CRLs) with prognostic value were screened. **(A)** Co-expression network of cuproptosis-related genes and CRLs by Pearson correlation analysis. **(B)** There were 49 CRLs found to have prognostic value by COX regression analysis with one variable. **(C)** Heat map of prognosis-related CRLs expression in COAD and normal tissues. **(D)** The expression of prognosis-related CRLs in COAD and normal tissues was shown in box plots. (* *P* < 0.05, ** *P* < 0.01, *** *P* < 0.001).

To identify lncRNAs associated with cuproptosis-related genes (CRGs), we performed co-expression analysis to reveal the correlation. Firstly, the co-expression network demonstrated the interaction between CRGs and CRLs (**Figure 1A**). Following that, using univariate COX regression analysis, 49 CRLs with prognostic value were identified (**Figure 1B**). The heat map and box plots indicated the expression difference of 49 CRLs between COAD and normal tissues (**Figures 1C, D**).

### Consensus clustering analysis

**Figure 2:**
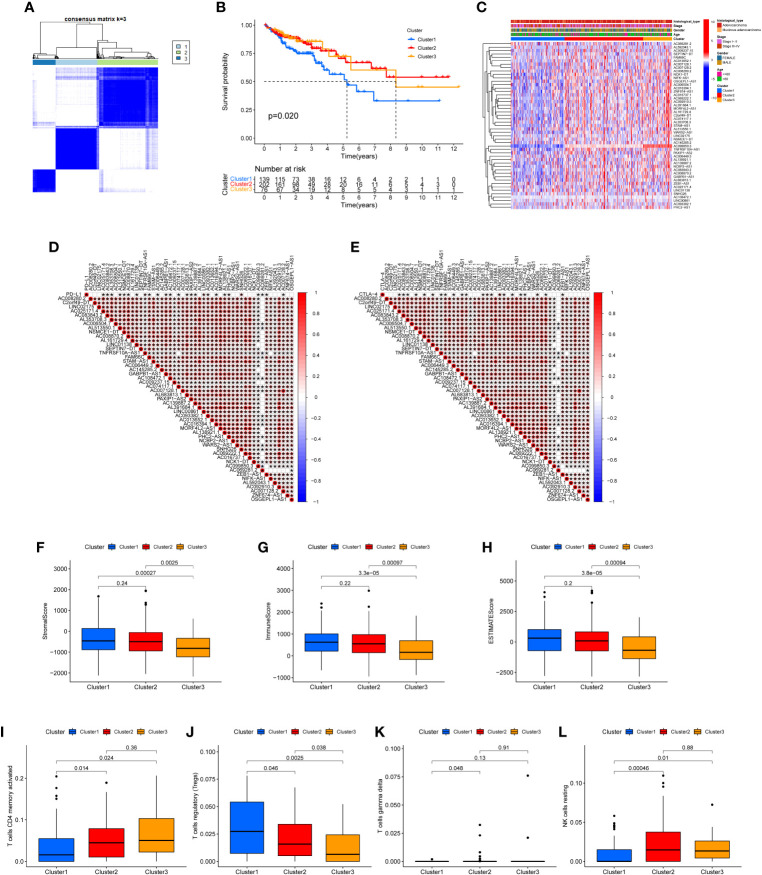
Clustering analysis *via* consensus. **(A)** When K = 3, the clustering was the most stable. **(B)** Survival analysis of the three clusters. The prognosis of Cluster 1 is the poorest. **(C)** A heat map depicting the differences in CRL expression and clinical features between the three clusters. **(D, E)** The immune checkpoints genes PD-L1 and CTLA-4, as well as prognosis-related CRLs, have a co-expression relationship. Stromal score **(F)**, Immune score **(G)**, ESTIMATE score **(H)**, the abundance of T cells CD4 memory activated **(I)**, T cells regulatory **(J)**, T cells gamma delta **(K)** and NK cell resting **(L)** in three clusters were shown.

The 49 CRLs were then put through a consensus clustering analysis to determine whether they might be used to stratify COAD patients. Based on the “ConsensusClusterPlus” program, a consensus cluster consisting of 49 CRLs was built (K represents cluster count, **Supplementary Figure 1**). The clustering exhibited the best stability when K = 3 based on the similarity of CRL expression levels and the proportion of fuzzy clustering measures (**Figure 2A**). As a result, 417 COAD patients were split into three clusters: cluster 1 (n = 139), cluster 2 (n = 202), and cluster 3 (n = 76). The prognosis of the three clusters was significantly different in survival analysis (*P*=0.020), with Cluster 1 having the worst prognosis (**Figure 2B**). In the form of a heatmap, **Figure 2C** depicted the differences in CRLs expression and clinical features between the three groups. The immune checkpoint genes PD-L1 and CTLA-4, as well as these lncRNAs, were found to have a co-expression relationship (**Figures 2C, D**). Following that, the analysis revealed the difference of Stromal score, Immunological score, ESTIMATE score, as well as the abundance of T cells CD4 memory activated, T cells regulatory, T cells gamma delta and NK cell resting in three clusters (**Figures 2E–L**).

### Construction and evaluation of the prognostic model

**Figure 3:**
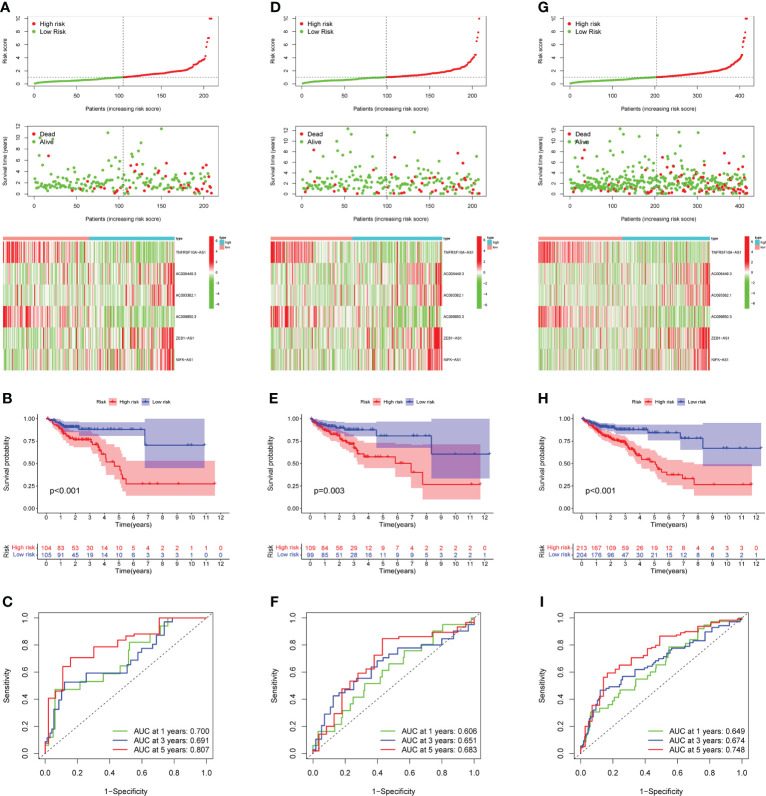
Prognostic model construction and evaluation. **(A)** In the training cohort, the patient with different risk score, survival status, and CRLs expression were shown. **(B)** Survival analysis of training cohort and the prognosis of high-risk group was significantly worse. **(C)** ROC curve revealed that in the training cohort, the AUC values for 1, 3, and 5-years OS were 0.700, 0.691, and 0.807, respectively. **(D, G)** In the testing and entire cohort, the patient’s risk score, survival status, and CRLs expression were shown. **(E)**The high-risk group’s prognosis in the testing cohort was also significantly worse. **(F)** In the testing cohort, AUC values of 1, 3, and 5-year OS were 0.606, 0.651, and 0.683, respectively, according to the ROC curve. **(H)** In the entire cohort, survival analysis revealed worse outcomes in the high-risk group. **(I)** In the entire cohort, the AUC values of the model in 1, 3, and 5-years OS were 0.649, 0.674 and 0.748, respectively.

The 49 CRLs described above were then further examined in order to reduce the scope and build a predictive model. COAD patients were separated into two cohort: training and validation (testing and entire). Univariate Cox regression analysis was performed on the training cohort, 12 CRLs (NSMCE1-DT, AL161729.4, LINC01138, SEPTIN7-DT, TNFRSF10A-AS1, AC006449.3, AC093382.1, PHC2-AS1, AC099850.3, AC069281.2, ZEB1-AS1, NIFK-AS1) with prognosis value were identified in COAD (**Table 1**). The LASSO Cox regression model was used to narrow the most robust lncRNAs for prognosis and build prognostic models in the training cohort. Ten-fold cross-validation was applied to overcome the over-fitting. To generate a prognostic CRLs signature model, multivariate Cox regression analysis was applied to evaluate the connection between CRLs and OS in the training set. The model is more stable when LAMDA = 6. (**Supplementary Figure 2**). TNFRSF10A-AS1, AC006449.3, AC093382.1, AC099850.3, ZEB1-AS1, and NIFK-AS1 were included in this model. The calculation formula is: Risk score =TNFRSF10A-AS1 * (-0.2449) + AC006449.3 * 1.407 + AC093382.1 *1.812 + AC099850.3 * (-0.0899) + ZEB1-AS1 * 0.4332 + NIFK-AS1 * 0.3956. The model was also tested in two validation cohort: testing, and the entire cohort. To begin, **Figure 3A** depicted the patient’s risk score, survival status, and six CRLs expression level in the training cohort. Patients were split into high-risk and low-risk groups based on median risk score, and survival analysis revealed that the high-risk group’s prognosis was significantly worse (**Figure 3B**, *P*<0.001). We further validated the expression of six CRLs in colorectal cancer cell lines. As shown in **Supplementary Figure 3**, the expressions of TNFRSF10A-AS1, AC099850.3, ZEB1-AS1 and NIFK-AS1 were significantly higher in tumor cells compared to those in FHC cells. Meanwhile, AC006449.3 expression was upregulated in HT-29 cells, but downregulated in HCT-116 cells. Analogously, AC093382.1 expression was significantly higher in Caco-2 and HT-29 cells but lower in HCT-116 cells (**Supplementary Figures 3A–F**). The AUC values for 1, 3, and 5 years in the training cohort were 0.700, 0.691, and 0.807 respectively, according to the results of ROC curve (**Figure 3C**). According to the same risk score calculation formula, different risk scores and survival status of patients in the testing and entire cohort were identified, and the difference of six CRLs expression level between the two risk groups was also analyzed (testing cohort, **Figure 3D**; entire cohort **Figure 3G**). In addition, the outcomes of patients with higher risk score in the validation cohort were significantly worse (**Figure 3E, H**). The 5-year AUC value of testing and entire cohort were 0.683 and 0.748 respectively, according to the ROC curve (**Figures 3F, I**). Moreover, risk score was found to be an independent prognostic factor for COAD patients in the above three cohorts using both univariate and multivariate COX regression (**Supplementary Figures 4A–F**). Survival analysis revealed the prognostic value of risk score in COAD patients with different ages, different stages, different genders and different histological types (**Supplementary Figures 5A–H**).

**Table 1:** Univariate Cox analysis generated 12 CRLs that are significantly related to the overall survival (OS).

lncRNA	HR	Lower 95% CI	Higher 95% CI	*P* value
NSMCE1-DT	18.1956	1.6439	201.3981	0.0180
AL161729.4	1.4211	1.0226	1.9749	0.0363
LINC01138	1.7057	1.0521	2.7654	0.0303
SEPTIN7-DT	13.2654	1.4138	124.4671	0.0236
TNFRSF10A-AS1	0.8024	0.6682	0.9635	0.0184
AC006449.3	7.1223	1.6987	29.8617	0.0073
AC093382.1	9.8668	2.0216	48.1573	0.0047
PHC2-AS1	2.5447	1.0419	6.2149	0.0404
AC099850.3	0.9164	0.8494	0.9886	0.0241
AC069281.2	1.5789	1.0899	2.2873	0.01573
ZEB1-AS1	2.3421	1.4098	3.8907	0.0010
NIFK-AS1	1.7646	1.0950	2.8437	0.0197

### Construction and evaluation of the nomogram

**Figure 4:**
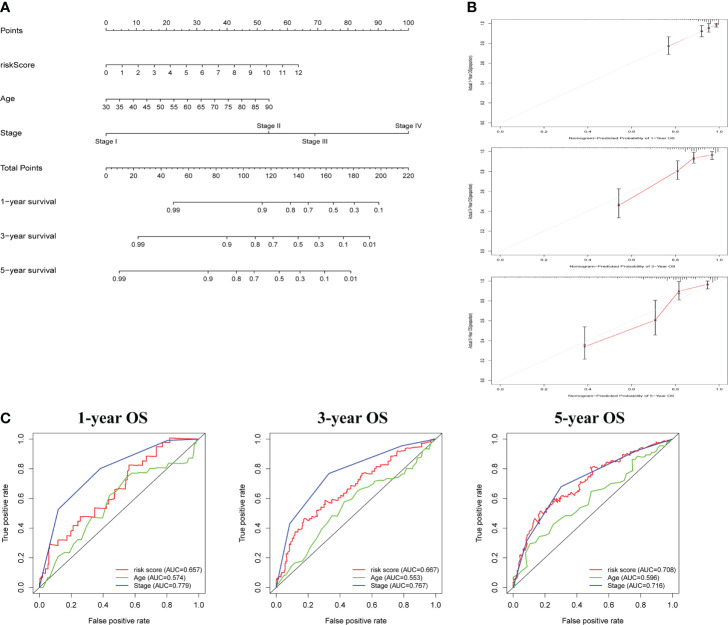
The construction and assessment of prognostic nomogram. **(A)** A nomogram for assessing 1-, 3-, and 5-year survival rates. **(B)**The calibration curves of the nomogram. **(C)**The ROC curve of risk score and clinical characteristics was investigated based on 1-, 3-, and 5-year OS.

Following that, nomogram was utilized to combine risk score and other clinical parameters to better evaluate the prognosis of COAD patients. We established a nomogram to assess COAD patients’ 1-, 3-, and 5-year survival rates (**Figure 4A**). The calibration curves revealed that the nomogram was accurate in predicting 1-, 3-, and 5-years survival rates (**Figure 4B**). Compared with the AUC value of the clinical features, risk score could be used to predict the OS of COAD patients (**Figure 4C**).

### Gene set enrichment analysis

**Figure 5:**
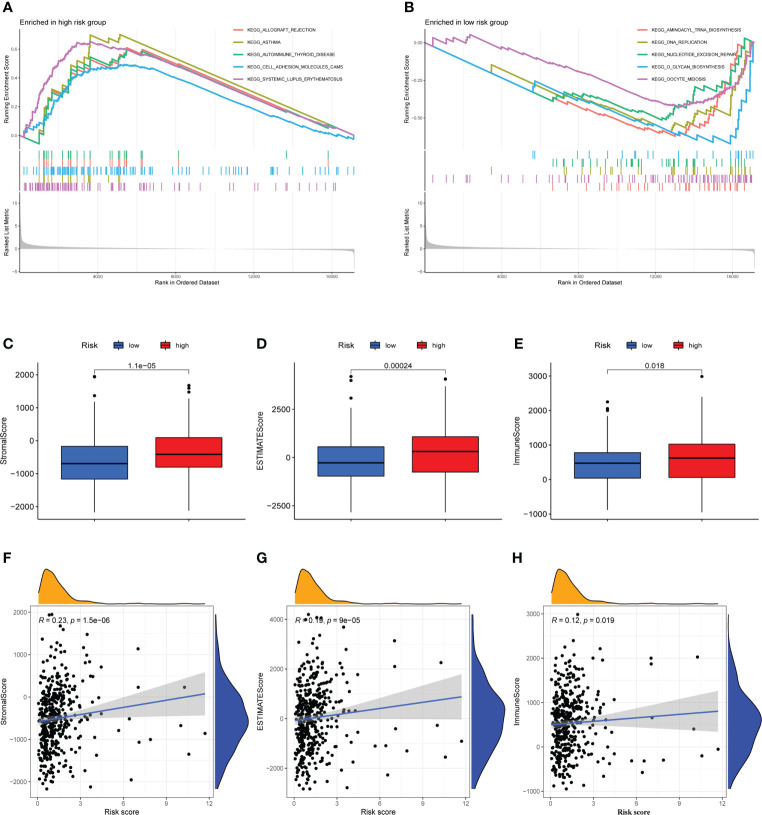
Immune microenvironment analysis and gene set enrichment analysis (GSEA). **(A)** Allograft rejection, asthma, autoimmune thyroid disease, cell adhesion molecules CAMs, and systemic lupus erythematosus are among the enriched KEGG pathways in the high-risk group. **(B)** Aminoacyl tRNA biosynthesis, dna replication, nucleotide excision repair, O-glycan biosynthesis, and oocyte meiosis are among the enriched KEGG pathways in the low-risk group. **(C–E)** The high-risk and low-risk groups had considerably different Stromal, ESTIMATE, and Immune scores, with the high-risk group having higher scores. **(F–H)** A Pearson correlation analysis revealed a strong positive relation between Stromal score, ESTIMATE score, Immune score and risk score.

GSEA was used to investigate the variations in pathway enrichment between high-risk and low-risk groups. Allograft rejection, asthma, autoimmune thyroid disease, cell adhesion molecules CAMs, and systemic lupus erythematosus were among the enriched KEGG pathways in the high-risk group (**Figure 5A**). However, aminoacyl tRNA biosynthesis, dna replication, nucleotide excision repair, O-glycan biosynthesis, and oocyte meiosis were among the enriched KEGG pathways in the low-risk group (**Figure 5B**). GSEA results revealed that patients with higher risk score were related to immune-related pathways while lower risk score patients were associated with tumor-related pathway, which maybe explained the survival different in two risk groups.

### Analysis of immune microenvironment

**Figure 6:**
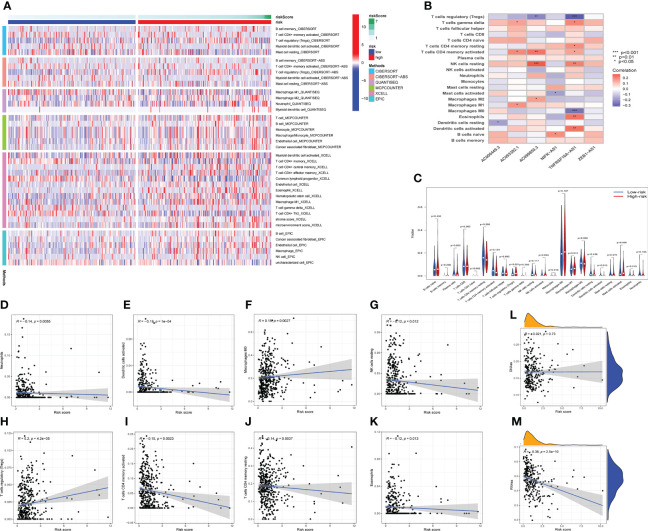
The immune landscape analysis. **(A)** The immune landscapes of high-risk and low-risk groups. **(B)** The correlation between CRLs and different immune cells, AC099850.3 had the highest positive correlation with resting NK cells, and TNFRSF10A-AS1 had the highest negative relation with T cells regulatory and macrophages M0. **(C)** The variations of immune cell infiltration levels between the high-risk and low-risk groups. **(D–K)** Analysis of the Pearson correlation between distinct immune cells and risk scores. **(L, M)** The correlation between DNA stem cell score (DNAss), RNA stem cell score (RNAss), and risk score. *P < 0.05, **P < 0.01, ***P < 0.001.

The occurrence and development of tumor are affected by immune microenvironment and the study of immune microenvironment can provide reference for immunotherapy of tumor. Stromal, Immune and ESTIMATE scores were significantly different between high-risk and low-risk groups, and may be higher in high-risk groups (**Figures 5C–E**). Pearson correlation analysis revealed a strong positive correlation between Stromal score, ESTIMATE score, and Immune score and risk score (**Figures 5F–H**). **Figure 6A** depicted the immunological landscape of high-risk and low-risk groups as heatmap, using CIBERSORT, QUANTISEQ, MCPCOUNTER, XCELL, and EPIC algorithms. Between the two groups, different amounts of immune cell infiltration were detected and subsequent correlation analysis revealed the relation between six CRLs expression and immune cells. Except ZEB1-AS1 expression has no correlation with immune cells, the other five CRLs have different degrees of correlation with immune cells, among which AC099850.3 had the highest positive correlation with resting NK cells and highest negative connection between TNFRSF10A-AS1 and Treg cells and macrophage M0 (**Figure 6B**). In the shape of a box diagram, **Figure 6C** depicted the differences in immune cell infiltration levels and the levels of infiltration of T cells regulatory and dendritic cells were found to be substantially different between the high-risk and low-risk groups (**Figure 6C**). A Pearson correlation analysis revealed the correlation between different immune cells and risk scores (**Figures 6D–K**). Finally, DNA stem cell score (DNAss) was shown to be unrelated to risk score (**Figure 6L**), however, RNA stem cell score (RNAss) was found to be significantly inversely associated to risk score (R= -0.38, P = 2.5E-10, **Figure 6M**).

### Expression analysis of m^6^A-related genes and human leukocyte antigen genes

**Figure 7:**
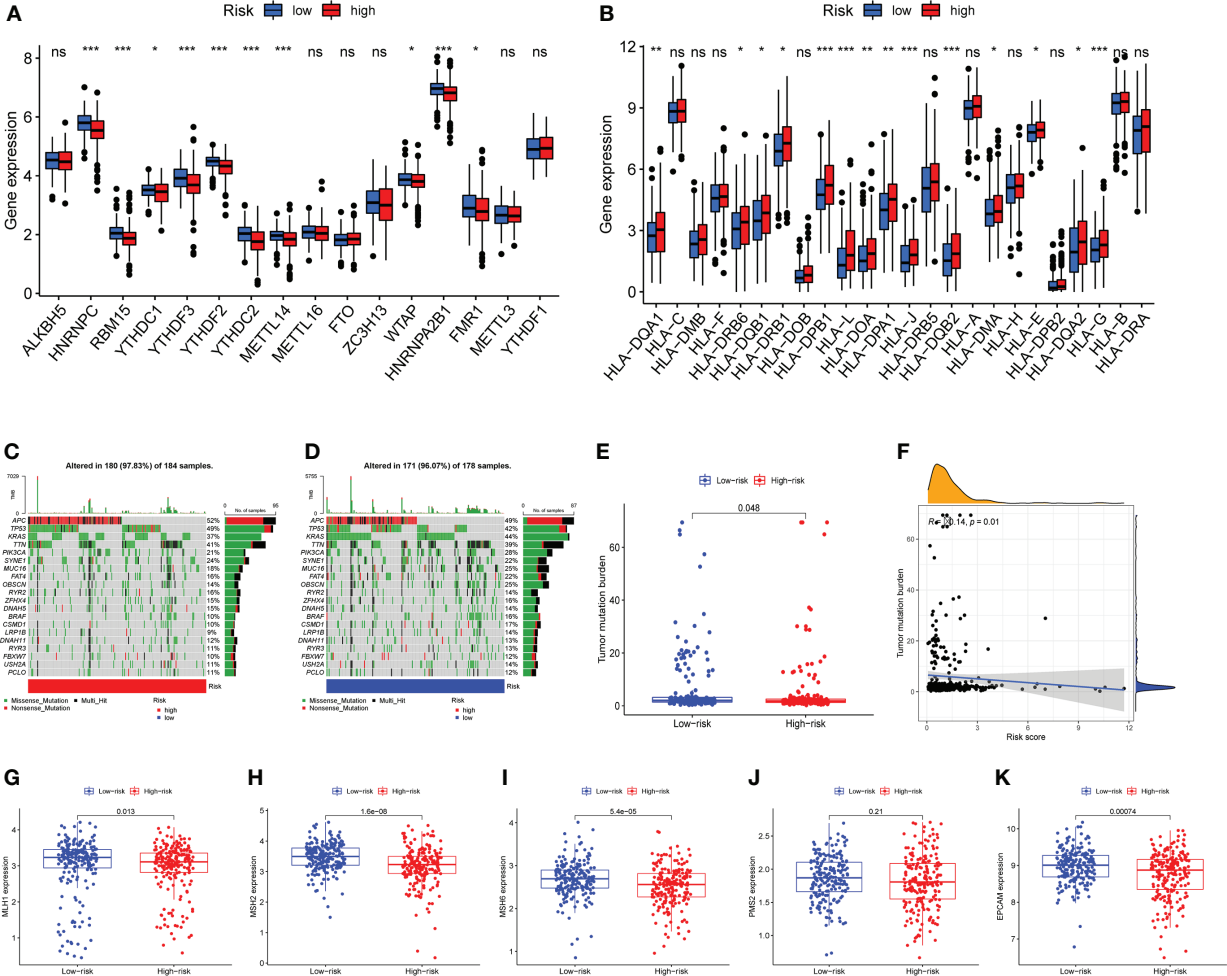
Expression analysis of m^6^A-related genes and human leukocyte antigen (HLA) related genes, mutation analysis. **(A)** Differential expression of m^6^A-related genes HNRNPC, RBM15, YTHDC1, YTHDF3, YTHDF2, YTHDC2, METTL14, WTAP, HNRNPA2B1, FMR1 were observed between the high-risk group and the low-risk groups. **(B)** The human leukocyte antigen gene analysis. HLA-DQA1, HLA-DRB6, HLA-DQB1, HLA-DRB1, HLA-DPB1, HLA-L, HLA-DOA, HLA-DPA1, HLA-J, HLA-DQB2, HLA-DMA, HLA-E, HLA-DQA2, and HLA-G were observed to be differentially expressed between high-risk and low-risk groups. **(C)** The mutation landscape of the high-risk group and draw the results into a waterfall diagram. **(D)**The waterfall diagram shows the mutation of patients in the low-risk group, and the mutation rate of APC was also highest. **(E)** Tumor mutation burden (TMB) analysis between the two risk groups. **(F)** Pearson-correlation analysis showed that there was a significant negative correlation between TMB and risk score. **(G–K)** Expression analysis of mismatch repair (MMR) protein. The expressions of MLH1, MSH2, MSH6 and EPCAM were significantly up-regulated in the low-risk group, while the expression of PMS2 showed no statistical difference between the two risk groups. ns, not significant,*P < 0.05, **P < 0.01, ***P < 0.001.

N^6^-methyladenosine (m^6^A) is a type of tumor epigenetics that plays an important function in tumor progression and human leukocyte antigen (HLA) has been related to tumor immunotherapy (43). Therefore, it is necessary to investigate the differences in expression of m^6^A-related genes and HLA-related genes between high-risk and low-risk groups. First, the expression of methylation-related genes HNRNPC, RBM15, YTHDC1, YTHDF3, YTHDF2, METTL14, WTAP, HNRNPA2B1, FMR1 was shown to differ between the high-risk and low-risk groups (**Figure 7A**). HLA-related genes including HLA-DQA1, HLA-DRB6, HLA-DQB1, HLA-DRB1, HLA-DPB1, HLA-L, HLA-DOA, HLA-DPA1, HLA-J, HLA-DQB2, HLA-DMA, HLA-E, HLA-DQA2, and HLA-G were shown to have varied levels of expression in high-risk and low-risk groups (**Figure 7B**).

### Mutation analysis

Mutations in numerous genes are associated with tumor formation, and the tumor mutation burden (TMB) is thought to be a helpful signal for evaluating immune checkpoint-related therapy. The mutation landscape of the high-risk group was depicted in **Figure 7C**. APC has the highest mutation rate, as can be observed. **Figure 7D** depicted the mutation landscape of the low-risk group, with APC having the highest mutation rate. TMB was also different across the two groups, with TMB being higher in the low-risk group (**Figure 7E**). A Pearson correlation study revealed that TMB and risk score had a substantial negative correlation (**Figure 7F**). Next, we focus on the expression of mismatch repair (MMR) protein, as it plays a key role in the process of COAD and is a major cause of gene mutations and microsatellite instability (MSI) (44). The expressions of MMR-related proteins MLH1, MSH2, MSH6, and EPCAM were substantially up-regulated in the low-risk group (*P*<0.05), while PMS2 expression did not differ statistically between the two risk groups (**Figures 7G–K**).

### Drug sensitivity analysis

**Figure 8:**
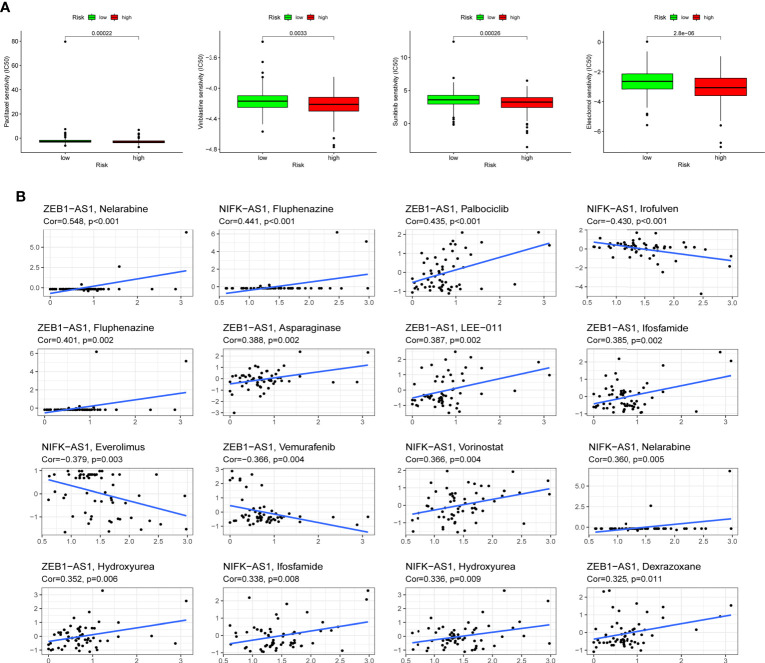
Drug sensitivity analysis. **(A)** Paclitaxel, Vinblastine, Sunitinib and Elescloml were observed to have lower IC50 values in the high-risk group, that is, the high-risk group was more sensitive to these drugs. **(B)** Scatter plot of correlation between CRLs expression and drug sensitivity.

Following the risk classification of COAD patients, medication sensitivity analysis can be used to identify effective treatments for different risk groups patients in order to individualized treatment. To begin with, paclitaxel, Vinblastine, Sunitinib, and Elescloml had lower IC50 values in the high-risk group, indicating that the patients with higher risk score were more responsive to these medications (**Figure 8A**). Following that, correlation analysis was used to identify medicines that were significantly correlated with the expression of CRLs. For instance, the analysis demonstrated that up-regulated ZEB1-AS1 expression was associated with increased drug sensitivity of tumor cells to nelarabine, palbociclib, fluphenazine, asparaginase, LEE-011, ifosfamide, hydroxyurea and dexrazoxane, while increased ZEB1-AS1 expression was related to the increased resistance to vemurafenib in COAD patients (**Figure 8B**).

## Discussion

COAD is still one of the most common cancer-related deaths in the world (45). Screening strategies, such as fecal occult blood test, screening colonoscopies and fecal immunochemical tests, can greatly reduce the incidence and mortality of COAD, but there are still many limitations of screening tests, and a large number of people eligible for screening miss the opportunity of screening (46, 47). With the innovations of risk stratification and development of personalized screening, the burden of COAD might be further reduced. At present, the treatment of advanced COAD remains a challenge due to stubborn drug resistance, metastasis and recurrence (48). Therefore, there is an urgent need to explore novel signatures for patients with COAD to assess prognosis, identify high-risk populations and guide personalized treatment. In recent years, regulatory cell death (RCD) has also been recognized as a promising target for cancers (49). Among them, cuproptosis is a copper-dependent and regulated new cell death mode, which is different from other known cell death regulation mechanisms (18). Further research on copper dependent cell death can provide a basis for the intervention of copper metabolism dysfunction related diseases and the potential application of anti-tumor. Therefore, cuproptosis may have complex crosstalk with metabolic reprogramming in cancers. While a number of RCD-related prognostic models have been developed to assess prognosis and immune microenvironment, this study is mainly report prognostic signature associated with cuproposis-related lncRNA (CRLs), which helps us understand the roles of cuproposis and CRLs in COAD.

Here, we performed a comprehensive bioinformatics analysis to explore the significance of CRLs in COAD, co-expression analysis and COX regression analysis were used to identify CRLs with prognostic significance. Subsequently, consensus cluster analysis showed that prognosis-related CRLs could divide patients with COAD into 3 clusters, which showed significant differences in prognosis and immune microenvironment. Following that, Lasso regression analysis was used to establish a prognostic signature with six CRLs. COAD patients could be separated into high-risk and low-risk groups according to median risk score, with the high-risk group having a much worse prognosis. This CRLs related signature gave a strategy of prognostic stratification for COAD patients. Finally, immune microenvironment, mutation and chemotherapeutic sensitivity analyses showed that this predictive signature could be used to provide evidence for immunotherapy and chemotherapy option.

Although screening and diagnosis of COAD have become more and more advanced, prognostic typing methods and sensitive genetic markers are still lacking (46, 50). Our study can provide CRLs-based prognostic model for patients with COAD, with an AUC value greater than 0.7 at 5 years OS, which can well classify patients into different prognostic groups, facilitate identification and early intervention of high-risk groups. There were also disparities in immune cell infiltration and TMB between high-risk and low-risk groups, providing some guidance for immunotherapy (51).

Our study identified six COAD prognostic markers correlated with cuproposis: TNFRSF10A-AS1, AC006449.3, AC093382.1, AC099850.3, ZEB1-AS1, and NIFK-AS1, which have been demonstrated to be associated with cancers in preliminary studies. First, Wei et al. discovered that TNFRSF10A-AS1 is a novel prognostic marker for colorectal cancer and may be related to autophagy (52). A regulatory network of lncRNA-miRNA-mRNA ceRNA was constructed for squamous cell carcinoma of tongue, and AC099850.3 was found to be strongly associated with the overall survival rate of patients (53). AC099850.3 has been confirmed to promote hepatocellular carcinoma (HCC) proliferation and invasion *via* the PRR11/PI3K/AKT axis and is a prognostic marker for HCC (54, 55). In addition, AC099850.3 was discovered as a predictive marker for non-small cell lung cancer (NSCLC) (56). Zinc finger E-box-binding homeobox 1 antisense 1 (ZEB1-AS1) facilitates the growth and metastasis of COAD cells, providing a new target for the diagnosis and treatment of COAD patients (57). Furthermore, ZEB1-AS1 can be used as one of the key lncRNAs in the construction of RCD-related prognostic signature (58). Consistent with previous studies, our study also included ZEB1-AS1 as the key lncRNA in RCD-related prognostic signature, which may reveal the important function of ZEB1-AS1 in RCD. Upregulation of NIFK-AS1 promote progression of HCC and Increased resistance to chemotherapy drugs through m6A methylation (59). Furthermore, NIFK-AS1 was discovered to suppress M2-like polarization of macrophages in endometrial cancer (60). However, there are currently few cancer research on AC006449.3 and AC093382.1, especially in COAD. In this study, we discovered a possible association between the six lncRNAs and cuproptosis, and offered evidences for their importance in the prognosis of COAD. Among six CRLs, TNFRSF10A-AS1 and AC099850.3 were protective factors while AC006449.3, AC093382.1, ZEB1-AS1 and NIFK-AS1 were adverse prognostic factors for COAD in this signature.

Immunotherapy, particularly immune checkpoint inhibitors, has been utilized to treat colorectal cancer in the past (61). However, “cold” tumors with low mutation rates and low microsatellite instability are not sensitive to immune checkpoint inhibitors (62, 63). As a result, it is critical to investigate the function of the predictive signature we developed in assessing mutation and expression of immune checkpoint-related genes in COAD. We discovered considerable disparities in tumor mutation burden (TMB), immune cell infiltration, HLA-related genes and mismatch repair proteins expression between the two risk groups based on signature constructed by cuproptosis-related lncRNAs, which might guide the immunotherapy for COAD patients. It also provided reference for understanding the potential association between tumor immunity and cuproptosis in colorectal cancer. Not only that, we identified more certain sensitive drugs for the COAD patients with higher risk score: Paclitaxel, Vinblastine, Sunitinib and Elescloml, which was conducive to the early intervention and precision treatment for COAD.

Previous bioinformatics studies have revealed the role of other types of CRD in COAD (64, 65). Cuproptosis, novel types of cell death, has not been explored in COAD and our study is the first to highlight the function of cuproptosis-related lncRNAs. These findings help us understand the interaction of many regulatory cell death patterns, and provide a reference for precise treatment of COAD. However, there are some limitations in our study. Although the mechanism of copper inducing cell death has similar markers and characteristics of different forms of RCD, cuproptosis has not been confirmed in cell death nomenclature (66–68). The AUC value of our signature is not very high, which is not greater than 0.8, and it may be limited by the sample size. And we lack relevant functional experiments to verify the function of cuproptosis-related genes and CRLs in the model, which will be improved in the future.

## Conclusions

Overall, our study is the first to develop a predictive signature based on the cuproptosis-associated lncRNA, providing a novel approach to risk stratification and potential biomarkers for COAD patients. This signature is valuable for assessing prognosis, immune infiltration and chemotherapy sensitivity, which may help provide guidance for detections and treatments in patients with COAD.

## Data availability statement

The datasets presented in this study can be found in online repositories. The names of the repositories and accession numbers can be found in the article / **Supplementary Material**.

## Author contributions

ZS and FW conceived the study and participated in the study design and performance. GC, and JL conducted the bioinformatics analysis and manuscript writing. CW, RG and MW revised the manuscript. All authors contributed to the article and approved the submitted version.

## Acknowledgments

We would like to extend our gratitude to the researchers and study patients for their contributions.

## Conflict of interest

The authors declare that the research was conducted in the absence of any commercial or financial relationships that could be construed as a potential conflict of interest.

## Publisher’s note

All claims expressed in this article are solely those of the authors and do not necessarily represent those of their affiliated organizations, or those of the publisher, the editors and the reviewers. Any product that may be evaluated in this article, or claim that may be made by its manufacturer, is not guaranteed or endorsed by the publisher.

## Supplementary material

Supplementary Figure 1 Consensus cluster analysis. **(A)** The cumulative distribution function (CDF) from *k* =2 to 9. **(B)** Relative change in area under CDF curve for *k* =2 to 9. **(C)** Tracking plot from *k* =2 to 9.

Supplementary Figure 2 The results of LASSO analysis. **(A)** The coefficient profile of 6 Cuproptosis-related lncRNAs. **(B)** 10-fold cross-validation of variable selection in LASSO models.

Supplementary Figure 3 The expression level of 6 Cuproptosis-related lncRNAs. **(A–F)** The expression level of TNFRSF10A-AS1, AC006449.3, AC093382.1, AC099850.3, ZEB1-AS1 and NIFK-AS1in colorectal cancer cell lines.

Supplementary Figure 4 Independent predictive factor analysis. Univariate and multivariate Cox regression analyses were used to evaluate whether risk score and clinical characteristics were independent predictors of COAD patients in three cohorts.

Supplementary Figure 5 Subgroup analysis of prognostic value of risk score. Survival analysis revealed the prognostic value of risk score in COAD patients with different ages **(A, B)**, different stages **(C, D)**, different genders **(E, F)** and different histological types **(G, H)**.
