# Supplementary figures and images for: Comprehensive analysis of the prognostic signature and tumor microenvironment infiltration characteristics of cuproptosis-related lncRNAs for patients with colon adenocarcinoma
Source: Front Oncol. 2022 Sep 23;12:1007918. doi: 10.3389/fonc.2022.1007918 (PMC9539748; doi:10.3389/fonc.2022.1007918)

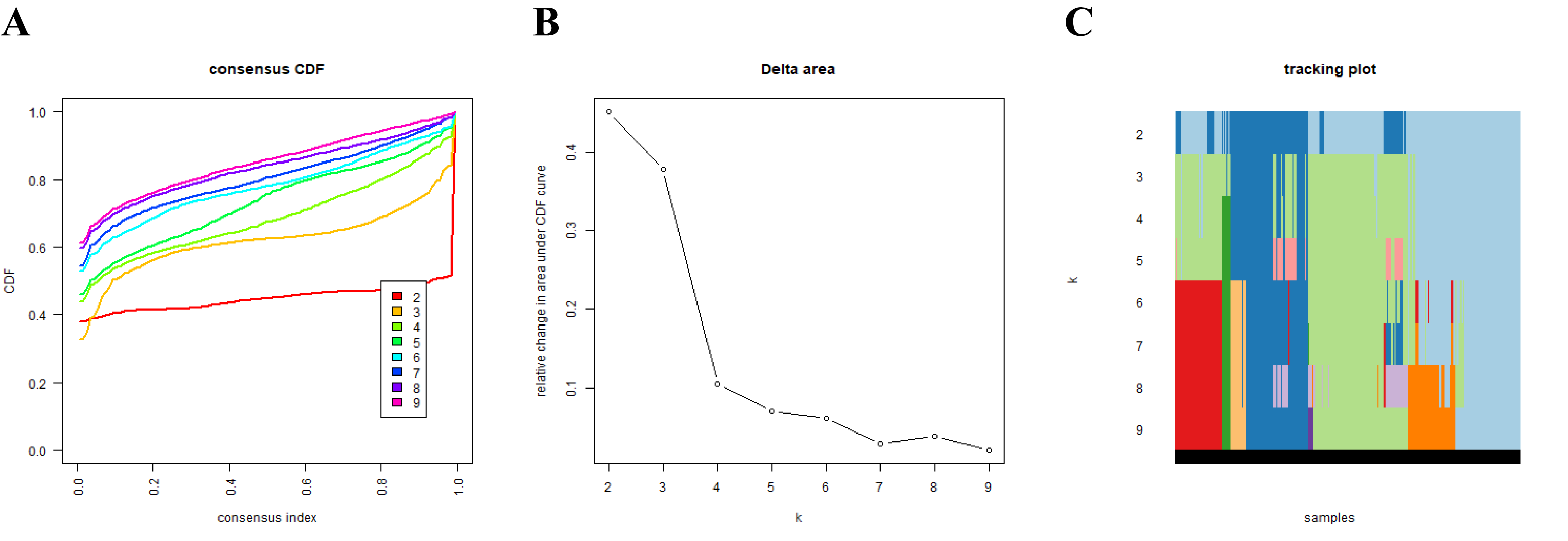

Supplement: Supplementary Figure 1 — Consensus cluster analysis. (A) The cumulative distribution function (CDF) from k =2 to 9. (B) Relative change in area under CDF curve for k =2 to 9. (C) Tracking plot from k =2 to 9. [file Image_1.tif]

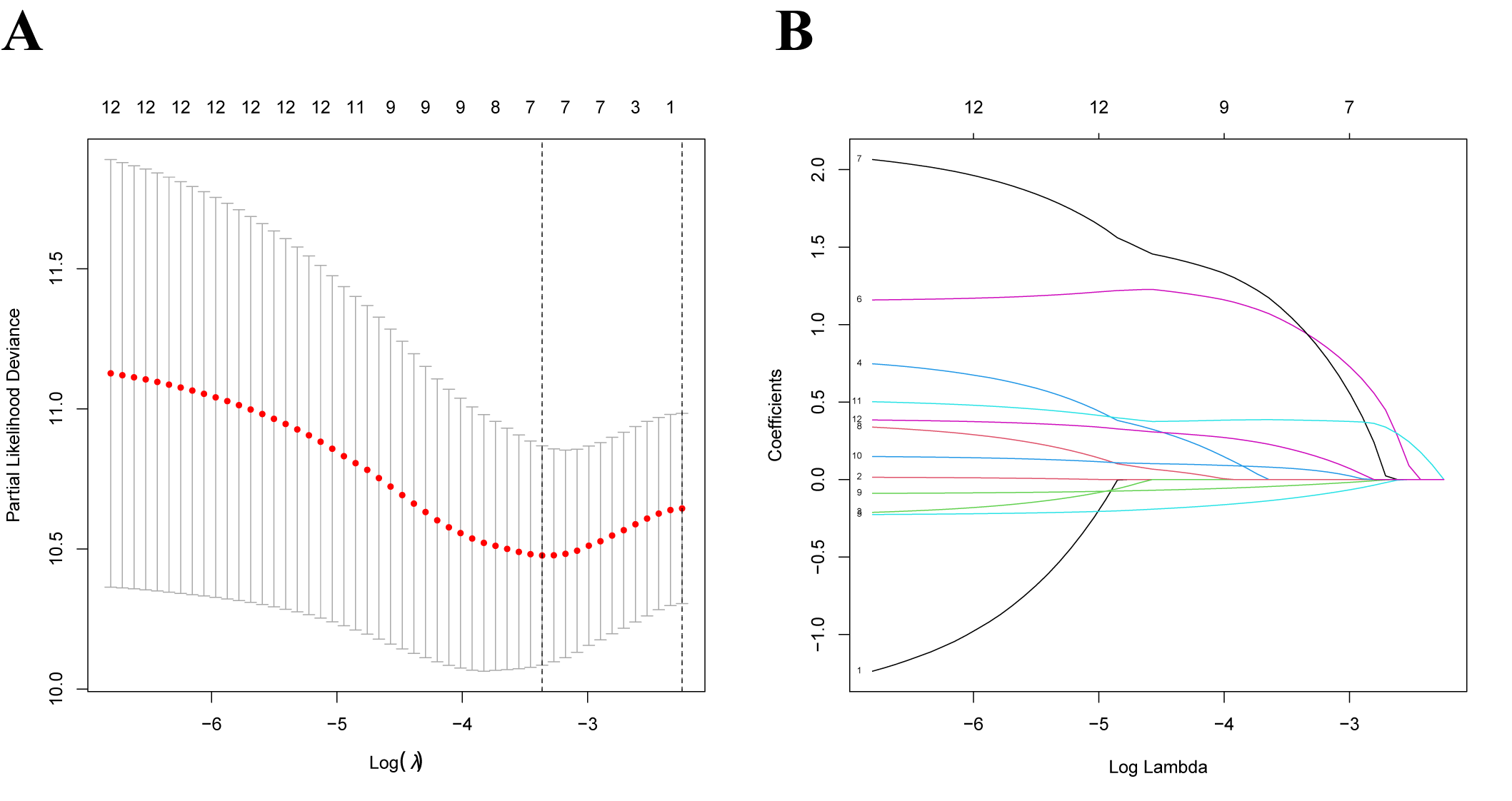

Supplement: Supplementary Figure 2 — The results of LASSO analysis. (A) The coefficient profile of 6 Cuproptosis-related lncRNAs. (B) 10-fold cross-validation of variable selection in LASSO models. [file Image_2.tif]

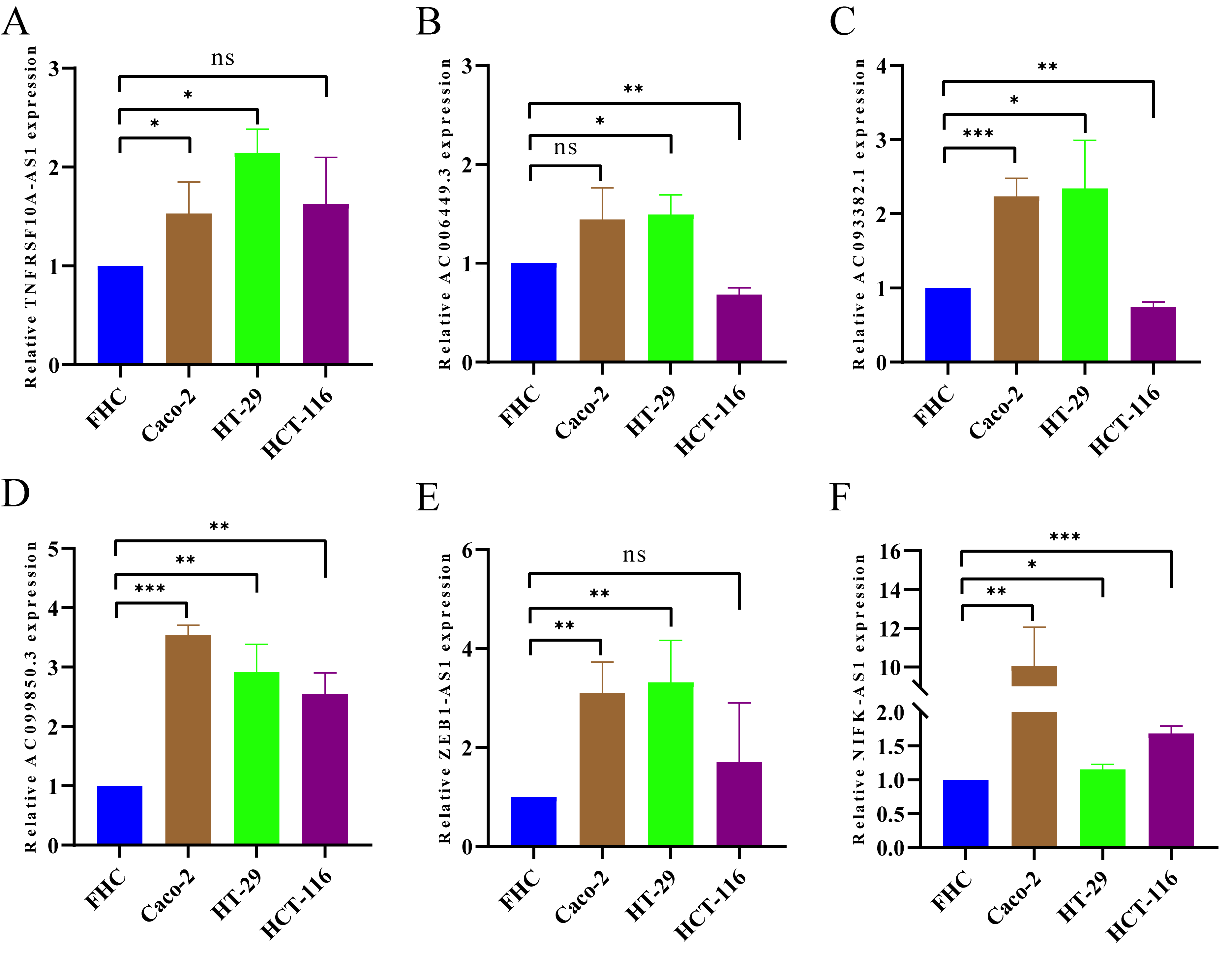

Supplement: Supplementary Figure 3 — The expression level of 6 Cuproptosis-related lncRNAs. (A–F) The expression level of TNFRSF10A-AS1, AC006449.3, AC093382.1, AC099850.3, ZEB1-AS1 and NIFK-AS1in colorectal cancer cell lines. [file Image_3.tif]

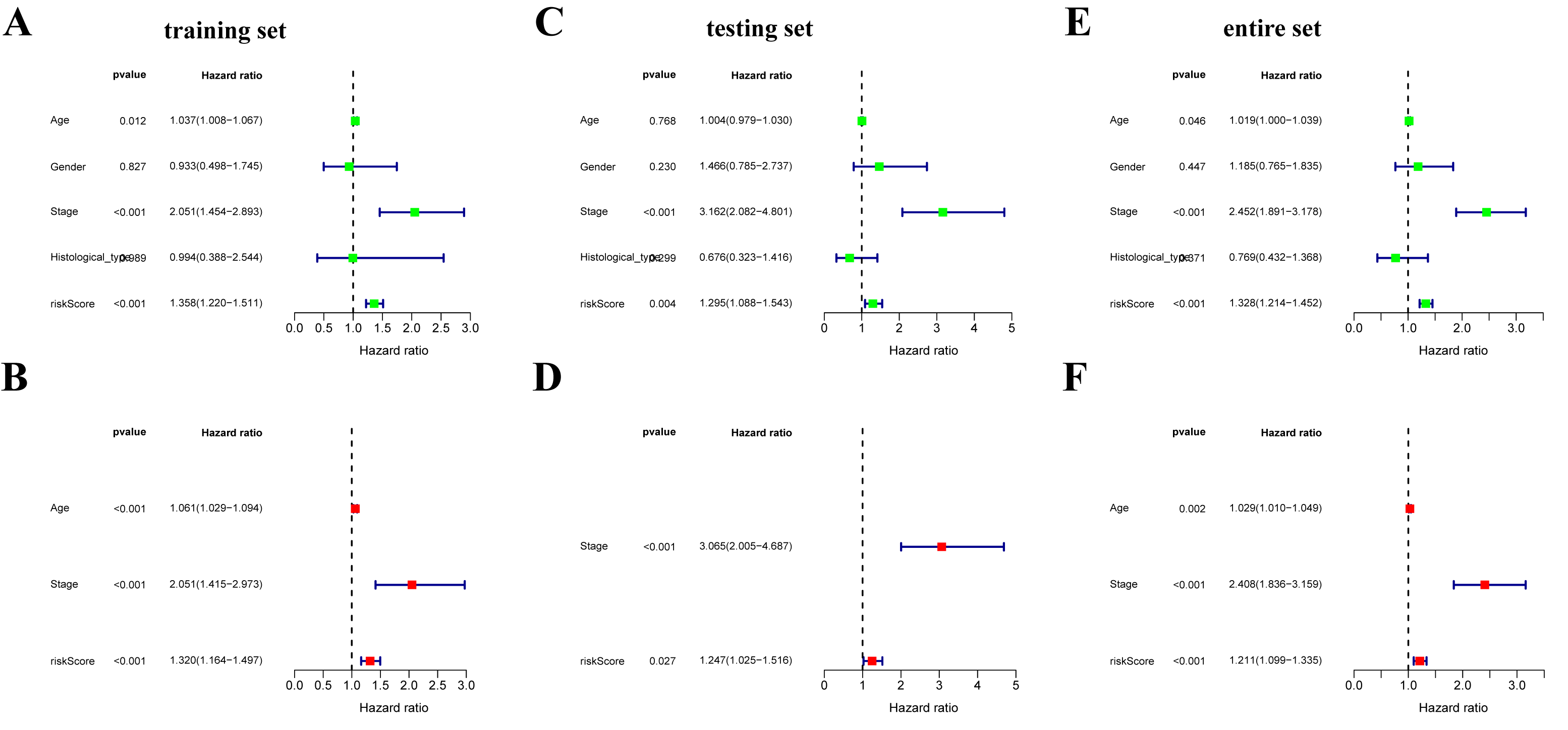

Supplement: Supplementary Figure 4 — Independent predictive factor analysis. Univariate and multivariate Cox regression analyses were used to evaluate whether risk score and clinical characteristics were independent predictors of COAD patients in three cohorts. [file Image_4.tif]

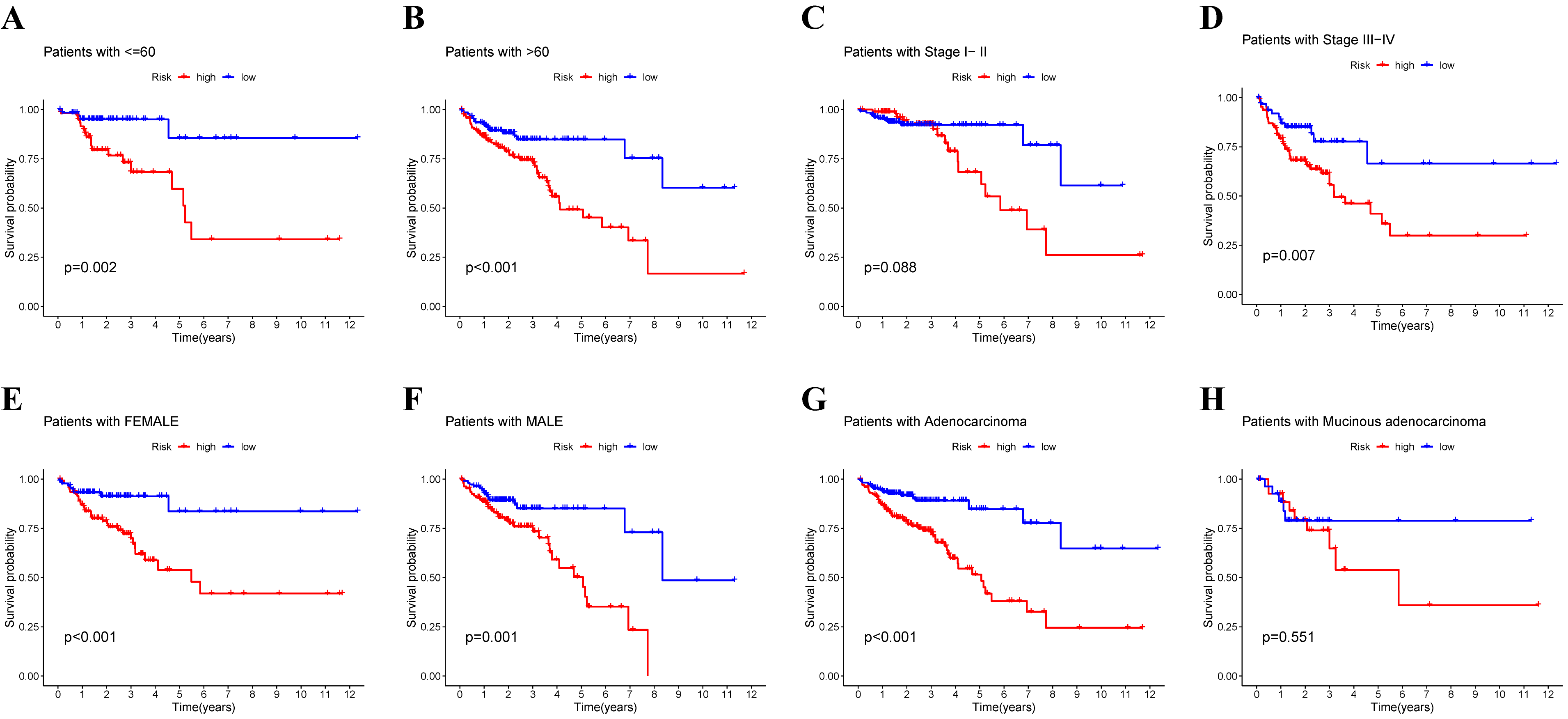

Supplement: Supplementary Figure 5 — Subgroup analysis of prognostic value of risk score. Survival analysis revealed the prognostic value of risk score in COAD patients with different ages (A, B), different stages (C, D), different genders (E, F) and different histological types (G, H). [file Image_5.tif]
