# Supplementary material for: Comprehensive analysis of the prognostic signature and tumor microenvironment infiltration characteristics of cuproptosis-related lncRNAs for patients with colon adenocarcinoma
Source: Front Oncol. 2022 Sep 23;12:1007918. doi: 10.3389/fonc.2022.1007918 (PMC9539748; doi:10.3389/fonc.2022.1007918)
Supplement: Supplementary file 6 [file DataSheet_1.docx]

| Covariates | Type | Entire | Testing | Training | *P* value |
| --- | --- | --- | --- | --- | --- |
| Age | <=65 | 175(41.97%) | 84(40.38%) | 91(43.54%) | 0.5798 |
|  | >65 | 242(58.03%) | 124(59.62%) | 118(56.46%) |  |
| Gender | FEMALE | 193(46.28%) | 98(47.12%) | 95(45.45%) | 0.8089 |
|  | MALE | 224(53.72%) | 110(52.88%) | 114(54.55%) |  |
| Stage | Stage I | 72(17.27%) | 38(18.27%) | 34(16.27%) | 0.1925 |
|  | Stage II | 160(38.37%) | 78(37.5%) | 82(39.23%) |  |
|  | Stage III | 117(28.06%) | 65(31.25%) | 52(24.88%) |  |
|  | Stage IV | 57(13.67%) | 22(10.58%) | 35(16.75%) |  |
|  | unknow | 11(2.64%) | 5(2.4%) | 6(2.87%) |  |
| T | T1 | 9(2.16%) | 6(2.88%) | 3(1.44%) | 0.1928 |
|  | T2 | 74(17.75%) | 38(18.27%) | 36(17.22%) |  |
|  | T3 | 284(68.11%) | 146(70.19%) | 138(66.03%) |  |
|  | T4 | 49(11.75%) | 18(8.65%) | 31(14.83%) |  |
|  | unknow | 1(0.24%) | 0(0%) | 1(0.48%) |  |
| M | M0 | 311(74.58%) | 161(77.4%) | 150(71.77%) | 0.0921 |
|  | M1 | 57(13.67%) | 22(10.58%) | 35(16.75%) |  |
|  | unknow | 49(11.75%) | 25(12.02%) | 24(11.48%) |  |
| N | N0 | 247(59.23%) | 123(59.13%) | 124(59.33%) | 0.824 |
|  | N1 | 98(23.5%) | 51(24.52%) | 47(22.49%) |  |
|  | N2 | 72(17.27%) | 34(16.35%) | 38(18.18%) |  |

**Table S1 Distribution of patients into training cohort, testing cohort and total cohort.**

**Table S2. Primer sequence of genes in qRT-PCR**

|  | Forward sequence | Reverse sequence |
| --- | --- | --- |
| TNFRSF10A-AS1 | TAGGATGAGAGCTGCCCACT | GGCCGTCCAGTAAGCTAAGG |
| AC006449.3 | TCCCCACCTCCTCCCTTC | GCACCTGACACCATGCCC |
| AC093382.1 | GAATGTTTCTTCCTTCTC | TTATTGCTCTTTCCTGAT |
| AC099850.3 | CCCAGGTTCAAGTAACTGGGAC | GACAATGCCTTGCCAAGGAATC |
| ZEB1-AS1 | AGGAATTCATGGCCTGTGGA | GGGGTAGGAATAGGGATAACTGT |
| NIFK-AS1 | TTCCCAAGAAGGTTCGATTG | TGCAGACTCCTGCTGTTGTT |
| β-ACTIN | CATGTACGTTGCTATCCAGGC | CTCCTTAATGTCACGCACGAT |
